# On the Potential of Bulk Metallic Glasses for Dental Implantology: Case Study on Ti_40_Zr_10_Cu_36_Pd_14_

**DOI:** 10.3390/ma11020249

**Published:** 2018-02-06

**Authors:** Alethea Liens, Aurélien Etiemble, Pascaline Rivory, Sandra Balvay, Jean-Marc Pelletier, Sandrine Cardinal, Damien Fabrègue, Hidemi Kato, Philippe Steyer, Tais Munhoz, Jerome Adrien, Nicolas Courtois, Daniel J. Hartmann, Jérôme Chevalier

**Affiliations:** 1 INSA-Lyon, MATEIS Laboratory, University of Lyon, UMR CNRS 5510, 20 Avenue Albert Einstein, 69621 Villeurbanne CEDEX, France; alethea.liens@insa-lyon.fr (A.L.); aurelien.etiemble@insa-lyon.fr (A.E.); pascaline.rivory@univ-lyon1.fr (P.R.); sandra.balvay@univ-lyon1.fr (S.B.); Jean-marc.pelletier@insa-lyon.fr (J.-M.P.); sandrine.cardinal@insa-lyon.fr (S.C.); damien.fabregue@insa-lyon.fr (D.F.); philippe.steyer@insa-lyon.fr (P.S.); tais.munhoz@insa-lyon.fr (T.M.); jerome.adrien@insa-lyon.fr (J.A.); daniel.hartmann@univ-lyon1.fr (D.J.H.); 2 Anthogyr SAS, 2237 Avenue A. Lasquin, 74700 Sallanches, France; n.courtois@anthogyr.com; 3 Institute for Materials Research, Tohoku University, 980-011 Sendai, Japan; hikato@imr.tohoku.ac.jp

**Keywords:** Ti-based bulk metallic glass, biomaterials, dental application, mechanical properties, biocompatibility

## Abstract

Ti_40_Zr_10_Cu_36_Pd_14_ Bulk Metallic Glass (BMG) appears very attractive for future biomedical applications thanks to its high glass forming ability, the absence of toxic elements such as Ni, Al or Be and its good mechanical properties. For the first time, a complete and exhaustive characterization of a unique batch of this glassy alloy was performed, together with ISO standard mechanical tests on machined implant-abutment assemblies. The results were compared to the benchmark Ti-6Al-4V ELI (Extra-Low-Interstitial) to assess its potential in dental implantology. The thermal stability, corrosion and sterilization resistance, cytocompatibility and mechanical properties were measured on samples with a simple geometry, but also on implant-abutment assemblies’ prototypes. Results show that the glassy alloy exhibits a quite high thermal stability, with a temperature range of 38 °C between the glass transition and crystallization, a compressive strength of 2 GPa, a certain plastic deformation (0.7%), a hardness of 5.5 GPa and a toughness of 56 MPa.√m. Moreover, the alloy shows a relatively lower Young’s modulus (96 GPa) than the Ti-6Al-4V alloy (110–115 GPa), which is beneficial to limit bone stress shielding. The BMG shows a satisfactory cytocompatibility, a high resistance to sterilization and a good corrosion resistance (corrosion potential of −0.07 V/SCE and corrosion current density of 6.0 nA/cm^2^), which may ensure its use as a biomaterial. Tests on dental implants reveal a load to failure 1.5-times higher than that of Ti-6Al-4V and a comparable fatigue limit. Moreover, implants could be machined and sandblasted by methods usually conducted for titanium implants, without significant degradation of their amorphous nature. All these properties place this metallic glass among a promising class of materials for mechanically-challenging applications such as dental implants.

## 1. Introduction

Titanium alloys have been traditionally used to design implants and implantable devices due to their excellent combination of high strength, high corrosion resistance and satisfactory biocompatibility [1]. However, these alloys seem to reach a plateau in terms of mechanical properties, which can be a limit as far as downsizing of implants is concerned in modern surgeries. For this reason, Ti-based Bulk Metallic Glasses (BMGs) have gained increasing attention during the past few decades. Ti-based bulk metallic glasses offer exceptional specific strength, high corrosion resistance, high hardness, low Young’s modulus, a certain amount of ductility (about 1%) and processing capabilities desired for biomedical applications [2]. Many Ti-based BMGs have been developed in the framework of Ti-Ni-Cu and Ti-Zr-Cu-Ni alloy systems [3,4,5,6,7] based on Inoue’s three empirical rules [8]. However, these Ti-based alloys contain a high amount of Ni or Be, which would prohibit their use for biomedical applications [9,10,11,12,13,14]. Recently, Ti-based bulk metallic glasses without toxic elements were proposed in the Ti-Zr-Cu-Pd system. They have demonstrated a high glass forming ability (with a critical diameter of 6 mm [15,16], which should be large enough to machine dental implants and dental abutment pieces), excellent mechanical properties [15] and a good corrosion resistance [16]. Therefore, the well-known Ti_40_Zr_10_Cu_36_Pd_14_ alloy shows a great potential to be used as a dental implant material, as it exhibits a higher strength and lower Young’s modulus (2 GPa and 90 GPa, respectively) compared to Ti-6Al-4V alloy [16,17,18].

Even if the main properties of Ti_40_Zr_10_Cu_36_Pd_14_ have already been measured in several papers [9,16,19,20,21,22,23,24], there is no complete set of data obtained on a single batch of material. For the first time, in this study, a complete characterization of the Ti_40_Zr_10_Cu_36_Pd_14_ alloy was therefore performed on one unique batch of material. Moreover, the overall fabrication (including machining and sandblasting, for example) of dental implants and the realization of ISO standard mechanical tests on implant-abutment assemblies made of metallic glasses have never been done to date, which is a crucial step to open the way to industrialization of this BMG as a dental material. Mechanical tests on real-sized dental and abutment assemblies were thus also performed following the ISO 14801-2008 standard and compared to medical-grade Ti-6Al-4V ELI alloy (ISO5832-3). They include both load to failure and fatigue tests. In summary, the aim of this study was to establish a complete multifunctional characterization of the Ti_40_Zr_10_Cu_36_Pd_14_ alloy’s behavior using the same batch, regarding especially mechanical tests on real-sized dental implants, cytocompatibility and resistance against sterilization, in order to complete the set of data available in the literature to assess the potential of this Ti-based BMG as a future biomaterial.

## 2. Results

### 2.1. Materials General Characterization

#### 2.1.1. Structural Analysis

**Figure 1:**
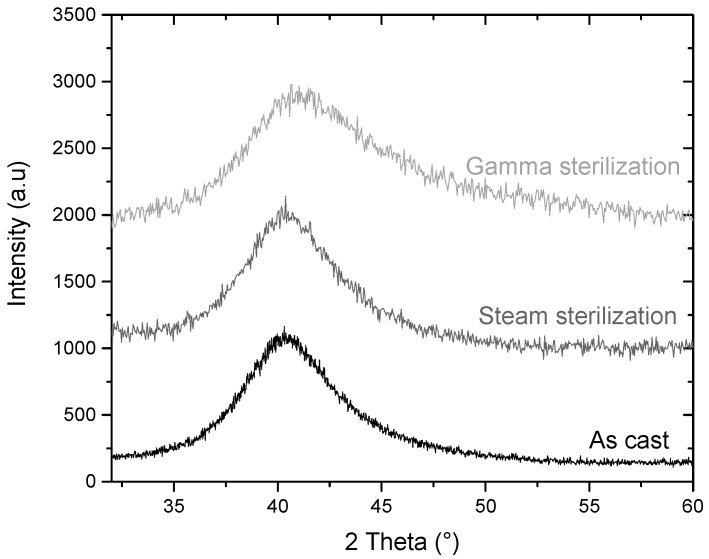
XRD pattern of the Ti_40_Zr_10_Cu_36_Pd_14_ BMG before and after steam or Gamma-ray sterilization (respectively 134 °C for 20 h or three doses at 40 kGy).

The chemical composition of the alloy, given in Table 1, corresponds well to the desired one. The amorphous state of the alloy is confirmed in Figure 1 as only one broad diffraction peak is observed in the as-cast state. The density was measured to be 7.08 g/cm^3^ (±0.02), which is consistent with the literature [25], and the fully-dense structure of the samples was confirmed by X-ray tomography.

#### 2.1.2. Thermal Stability

**Figure 2:**
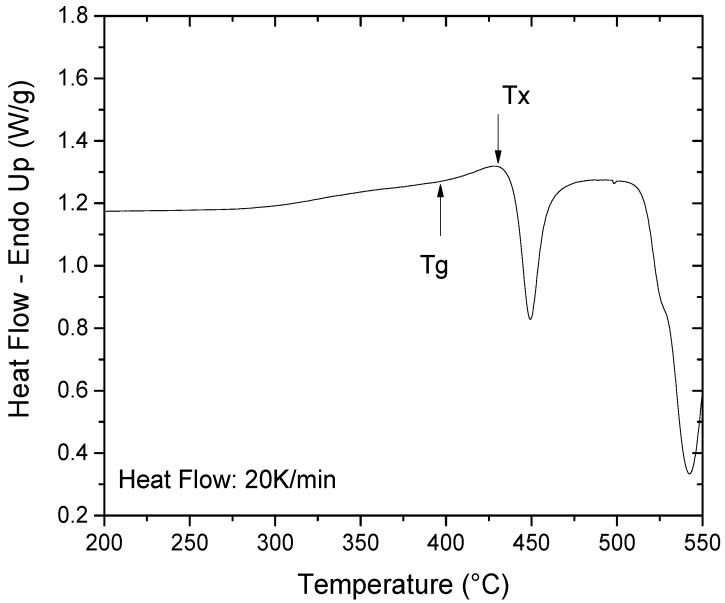
DSC scan of the Ti_40_Zr_10_Cu_36_Pd_14_ BMG.

The glass transition temperature (Tg), onset temperature of crystallization (Tx) and super-cooled liquid region (ΔTx = Tx − Tg) can be seen on the DSC scan shown in Figure 2 and are summarized in Table 1. The results are consistent with values published in the literature [15,16,19,20,26].

**Figure 3:**
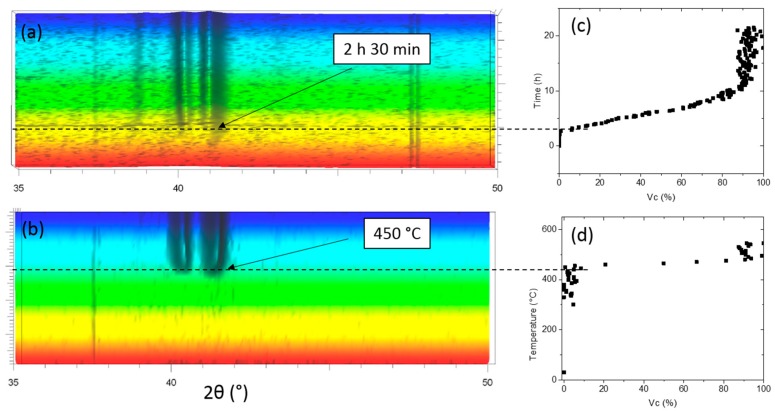
In situ X-ray diffraction pattern of Ti_40_Zr_10_Cu_36_Pd_14_ BMG during both isothermal annealing at 420 °C (**a**) and continuous heating to 550 °C (**b**) and evolution of the calculated volume fraction of crystalline particles as a function of time (**c**) or temperature (**d**).

Figure 3 shows the XRD patterns obtained after both continuous heating from room temperature to 550 °C (a) and isothermal annealing at 420 °C (b). Regarding the continuous heating, the alloy is initially fully amorphous (only a very broad hump is observed). At 450 °C, peaks appear and grow with time until saturation. The crystallization temperatures measured by both XRD (450 °C) and DSC (432 °C) technics are consistent, the slight difference being explained by the different heating rates [27]. It may be proposed that the first crystalline phases are PdTi_2_, CuTi and Cu_8_Zr_3_, as shown in Table 1, which is again in agreement with expected phases found in the literature [15,16,21,25,28].

To estimate the thermal stability of the amorphous structure, the alloy was heated at 420 °C for 20 h. This temperature was chosen as it is close to the crystallization temperature [27,29]. After an incubation time of 2.5 h, a continuous increase of the volume fraction of the crystalline phase is observed, and then, a plateau is reached when the crystallization is completed after about 11 h.

To estimate the evolution of the volume fraction of crystalline domains, peak areas were first evaluated in the range 2θ ϵ {39.7°–41.8°} and the amorphous phase fraction *X_am_* calculated by:

(1) $$X_{am}=\frac{I_{SC}-I_{C}}{I_{am}-I_{C}}$$

(2) $$X_{C}=1-X_{am}$$

where *I_SC_* is the area of the amorphous background in this 2θ range, measured at a defined time (or temperature), *I_am_* the area of the amorphous background for the 100% amorphous alloy and *I_C_* the area of the background (experimental error) for the fully-crystalline alloy. *X_C_* is then the volume fraction of the crystalline phase.

The results are reported as a function of time (at 420 °C) and temperature (during continuous heating) in Figure 3c,d.

A grain size evaluation has been conducted following the Debye–Scherrer equation (Equation (3)). The size of the crystallites formed is given by:

(3) $$D=\frac{K\times \lambda }{H\times \cos\theta }$$

with *D* the diameter of the crystallites, *K* a constant equal to 0.9, *λ* the wavelength (1.54 Å in our case), *H* the width at half maximum and *θ* the position of the peak (results gave here similar values for both peaks). After a quite rapid evolution, the grain size saturated rapidly to 19 nm, indicating a fast nucleation and low growth rate.

#### 2.1.3. Mechanical Characterization

The ultrasonic measurements of the Ti_40_Zr_10_Cu_36_Pd_14_ BMG’s Young’s modulus and Poisson’s ratio are gathered in Table 2. Results are consistent with the literature [19,23,30,31]. Vickers hardness values are also summarized in Table 2. As can be seen, the alloy exhibits a very high hardness value (556 HV), already reported in the literature [23,32,33].

**Figure 4:**
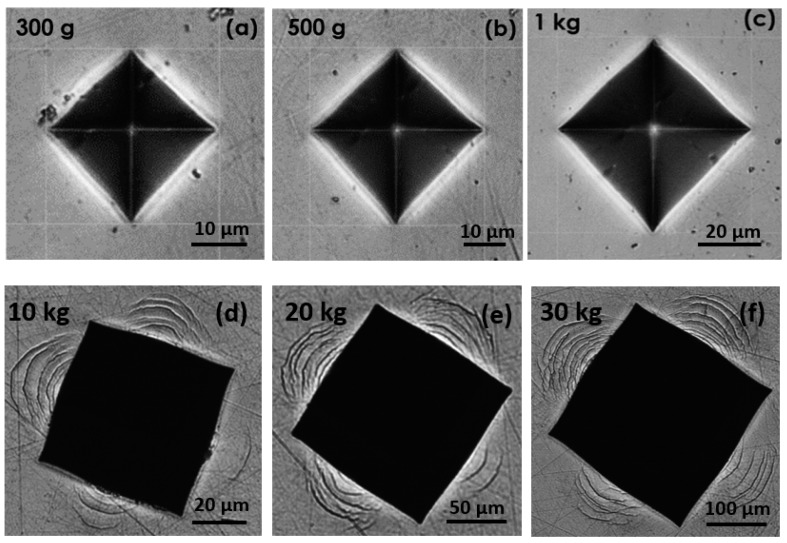
Optical microscopy observations of the Vickers indents of the Ti_40_Zr_10_Cu_36_Pd_14_ BMG after indentation at: (**a**) 300 gram—force (gf); (**b**) 500 gf; (**c**) 1 kgf; (**d**) 10 kgf; (**e**) 20 kgf and (**f**) 30 kgf.

The indent shapes and morphologies observed by optical microscopy are shown in Figure 4. The alloy does not show any crack or shear band for loads lower than 10 kgf. However, shear bands, characteristic of local plasticity, start to appear at 10 kilogram—force (kgf) (d). Then, the number of shear-bands increases with the applied load as shown in Figure 4. However, even at a very high load of 30 kgf (f), no cracks were observed.

**Figure 5:**
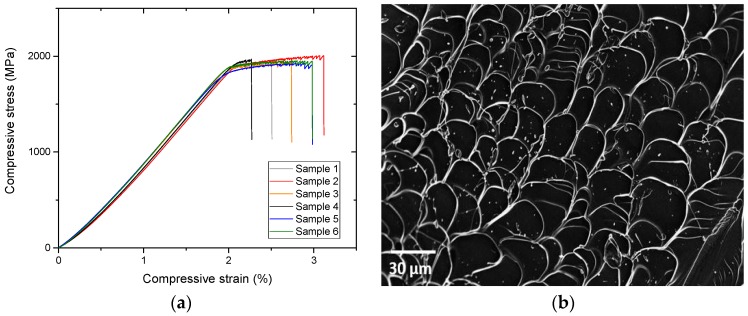
(**a**) Compressive stress-strain curve of the Ti_40_Zr_10_Cu_36_Pd_14_ metallic glass rod with a diameter of 3 mm and a height of 5 mm; (**b**) SEM image of the fracture surface of the Ti_40_Zr_10_Cu_36_Pd_14_ metallic glass rod subjected to fracture by the compression test.

Figure 5a shows the compressive stress-strain curves of the BMG. Mechanical properties (compressive strength, elastic and plastic strains and specific strength) deduced from the curves are summarized in Table 2. The alloy exhibits an elastic strain of about 2%, followed by a small-serrated plastic elongation of about 0.7%, indicating that the cast alloy possesses a certain ductility. The average compressive strength is 2 GPa, with a high reproducibility. As a result, the specific strength, which is the ratio of strength to density of the alloy, is calculated to be 2.8 × 10^5^ N∙m/kg. This value is consistent with the one obtained by S.L. Zhu et al. [15] and higher than the specific strength of biomedical titanium alloys (2 × 10^5^ N∙m/kg). However, an important dispersion in the strain values is observed between the six tested alloys (Figure 5).

Fractographic observations were carried out to examine the fracture mode of the BMG. The sample was fractured into two segments under compressive stress, and the fracture appears to take place along the maximum shear stress plane, with an angle of approximately 45° to the direction of the applied compressive load. The dominant fractographic feature is a smooth and well-developed vein pattern, typical of metallic glasses, as shown in Figure 5b.

### 2.2. Characterization for a Future Biomedical Application

#### 2.2.1. Resistance to Sterilization

As seen Figure 1, the alloy after steam sterilization does not show any crystalline peak and only displays a broad diffraction peak, as for the as-cast sample. Therefore, 20 h at 134 °C in water steam under 2 bars (simulating approximately 20 cycles of sterilization) do not change the amorphous nature of the Ti_40_Zr_10_Cu_36_Pd_14_ BMG. The same conclusion is drawn with gamma sterilization, since three doses of 40 kGy of gamma rays do not affect the amorphous nature of the material, as observed in Figure 1.

#### 2.2.2. Corrosion Resistance

**Figure 6:**
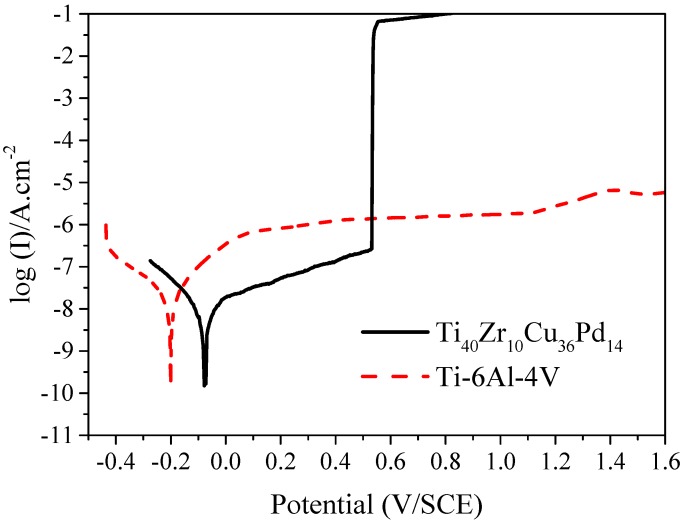
Anodic and cathodic polarization curves of the Ti_40_Zr_10_Cu_36_Pd_14_ BMG compared to the Ti-6Al-4V, in saline solution.

The potentiodynamic curves of the Ti_40_Zr_10_Cu_36_Pd_14_ BMG and the Ti-6Al-4V alloy obtained in 0.9% NaCl solution are shown in Figure 6. The corrosion potential (*E_corr._*) and the corrosion current density (*I_corr._*) extracted from the polarization curves, as well as the polarization resistance (*R_p_*) measured at the end of the preliminary Open Circuit Potential (OCP), are summarized in Table 2. Corrosion potential and current density are used to evaluate respectively the driving force for corrosion and the corrosion reaction kinetics. A passivation plateau can be observed in Figure 6 for both BMG and reference alloy. Pitting was also observed for the metallic glass at around 500 mV/SCE.

**Figure 7:**
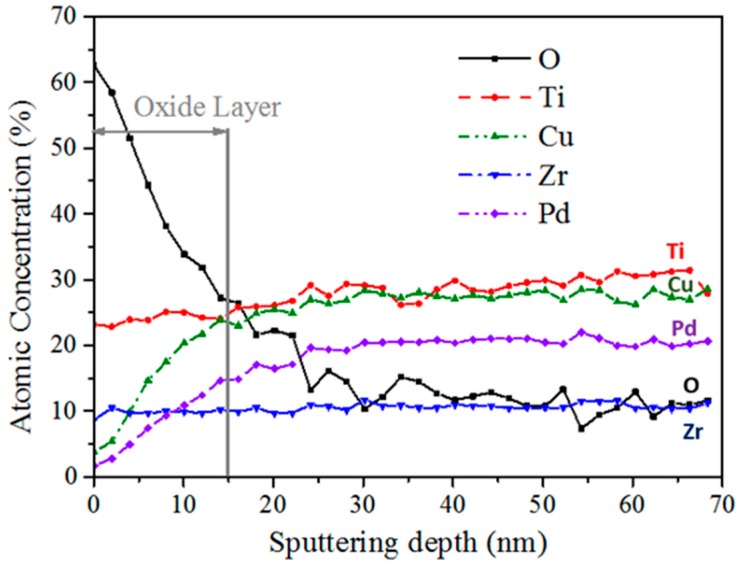
Atomic concentration profiles of the different elements composing the oxide film at the surface of the Ti_40_Zr_10_Cu_36_Pd_14_ metallic glass characterized by XPS.

Atomic concentration profiles of Ti, Zr, Cu, Pd and O along the depth at the surface of the Ti_40_Zr_10_Cu_36_Pd_14_ as-cast BMG are shown in Figure 7. A superficial oxygen enrichment is recorded in the first 15-nanometer depth, associated with an increase in both Ti and Zr contents (when related to Cu and Pd). This oxide layer should correspond to a protective passive layer, on the basis of the low corrosion rate of the BMG. It was also observed that the Ti2p3/2 Auger peak, initially associated with TiO_2_ at 458 eV at the extreme surface, progressively shifted to the metallic form at 454 eV as the depth increases. Similarly, for the zirconium, a progressive evolution, from the oxide ZrO_2_ (Zr3d5/2 peak centered at 182.2 eV) to the zirconium metal Zr0 (Zr3d5/2 peak centered at 179 eV) in the Ti_40_Zr_10_Cu_36_Pd_14_ BMG, was observed. Moreover, a low content of copper oxide Cu_2_O was also found at the passive layer surface (Auger peak centered at 569.8 eV).

#### 2.2.3. Cytocompatibility

**Figure 8:**
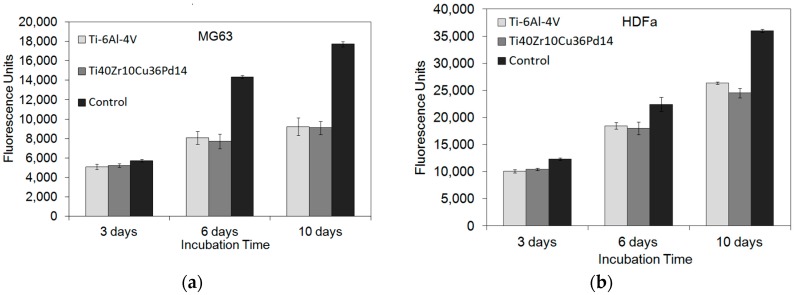
Cell viability proliferation measured by the PrestoBlue method on osteoblast MG63 and fibroblast Human Dermal Fibroblasts (HDFa) cultured on Ti_40_Zr_10_Cu_36_Pd_14_ BMG and Ti-6Al-4V after 3, 6 and 10 days with a control on the culture disk surface. (**a**) MG63; (**b**) HDFa.

Figure 8 displays the dependency of cell viability on incubation periods for both osteoblast-like MG63 cells and fibroblast Human Dermal Fibroblasts (HDFa) for both the Ti_40_Zr_10_Cu_36_Pd_14_ BMG and Ti-6Al-4V alloy. Whatever the material and the type of cells, the measured fluorescence, proportional to the quantity of cells, gradually increases with time. In addition, statistical *t*-tests reveal that no statistical differences were obtained between the two materials, indicating that the cells’ viability is comparable for the Ti_40_Zr_10_Cu_36_Pd_14_ BMG and the Ti-6Al-4V alloy for osteoblast MG63, as well as fibroblast HDFa.

**Figure 9:**
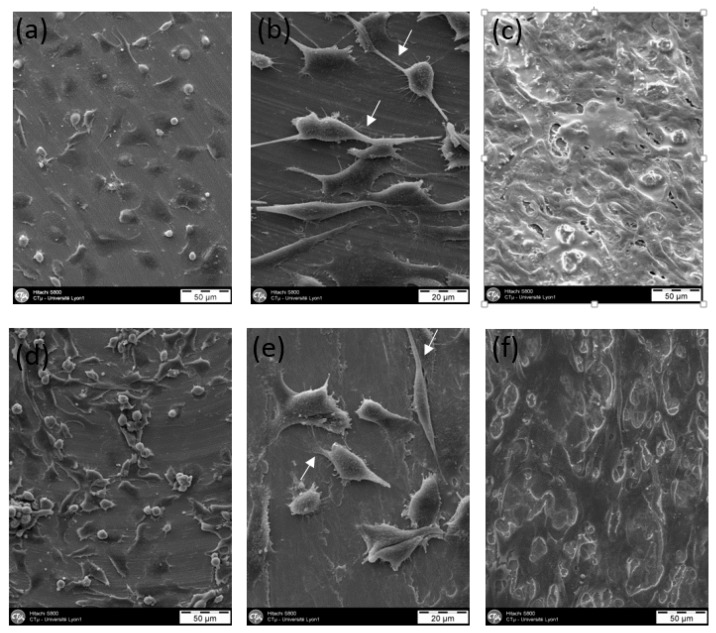
Morphologies of MG63 cells cultured on Ti-6Al-4V alloy for 6 h (**a**), 24 h (**b**) and 3 days (**c**) and on Ti_40_Zr_10_Cu_36_Pd_14_ BMG for 6 h (**d**), 24 h (**e**) and 3 days (**f**).

SEM images of the MG63 osteoblast-like cells attached on the Ti_40_Zr_10_Cu_36_Pd_14_ BMG and Ti-6Al-4V metal substrates after seeding for 6 h, 24 h and 3 days are shown in Figure 9. It can be seen that the MG63 cells adhered and spread out on the two materials’ surface. They show a conformal morphology, and at day 10 (D10), there is the presumption of extracellular matrix synthesis for the two materials.

#### 2.2.4. Tests on Dental Implants

**Figure 10:**
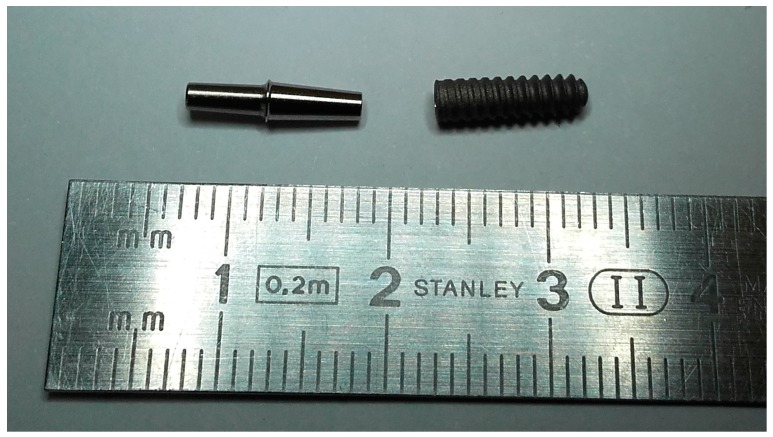
Image of the implant-abutment assembly prototype made of Ti_40_Zr_10_Cu_36_Pd_14_ BMG, machined in the same conditions as Ti-6Al-4V Extra Low Interstitial (ELI) assemblies.

Figure 10 shows an image of one of the BMG machined implant-abutment assemblies made of the Ti_40_Zr_10_Cu_36_Pd_14_ BMG following the same machining conditions as the ones of Ti-6Al-4V assemblies.

**Figure 11:**
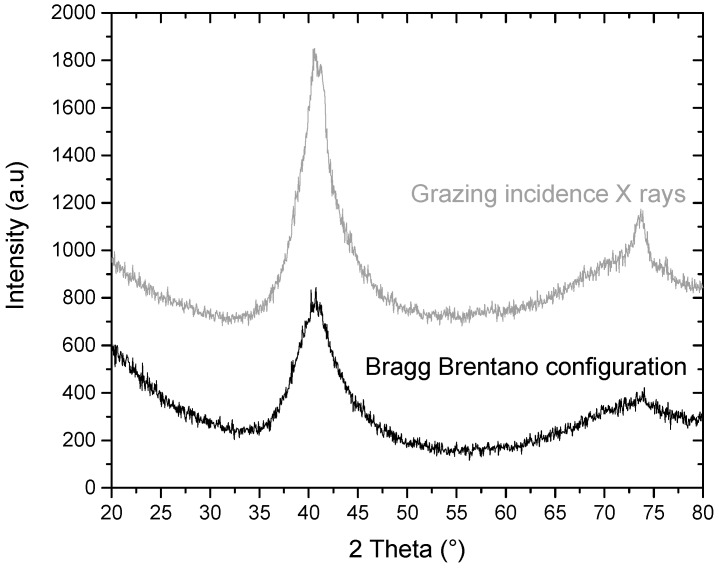
XRD pattern of a machined abutment with both X-rays configurations: grazing incidence X-rays and Bragg–Brentano configuration.

Figure 11 represents both the grazing incidence X-ray patterns obtained at the surface of an abutment after machining (X-ray penetration depth of roughly 1.4 µm) and a standard Bragg–Brentano pattern (X-ray penetration depth for 2θ = 75° of roughly 9 µm).

With grazing incidence XRD, peaks, attributed to the formation of crystalline phases, are present. They were also identified after the full crystallization of the alloy after in situ XRD as shown previously in this study. As shown by other authors [15,16,21,25,28], these peaks can correspond to a mixture of intermetallic compounds such as CuTi, PdTi_2_ and Cu_8_Zr_3_. The presence of these intermetallic compounds confirms that the final mechanical treatments of the alloys lead to a thin crystallized layer at the surface and that the observed peaks are not linked to a possible oxidation of the surface.

However, the same piece analyzed with the Bragg–Brentano configuration exhibits a broad peak, typical of an amorphous structure.

Optical interferometry measurements reveal that the roughness parameter Sa of the metallic glass after sandblasting and acid washing (1.3 µm) was slightly lower than the one of Ti-6Al-4V alloy after the same surface treatments (2.1 µm). This result is not surprising as the hardness of the metallic glass is higher than that of Ti-6Al-4V alloy (556 HV_1_ and 340 HV_1_, respectively), making the amorphous alloy less sensitive to surface treatments and mechanical abrasion.

**Figure 12:**
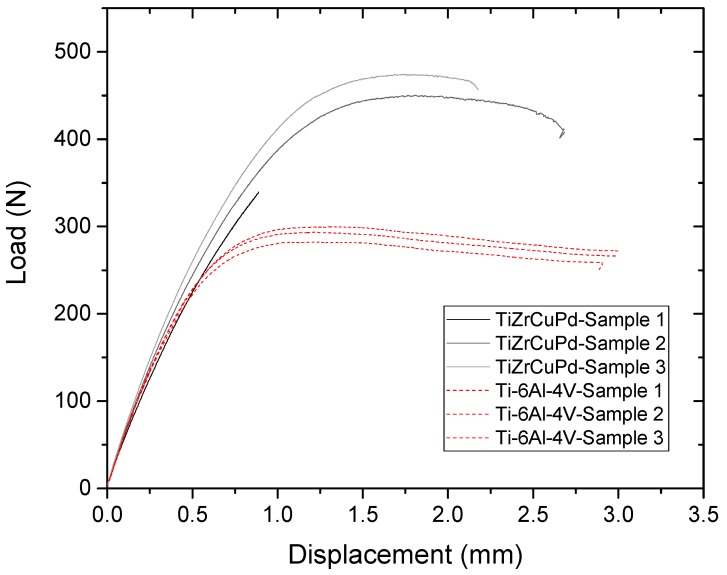
Curves of the load as function of the displacement for both Ti_40_Zr_10_Cu_36_Pd_14_ BMG and Ti-6Al-4V alloy after a test to failure on the prototype dental implants.

**Figure 13:**
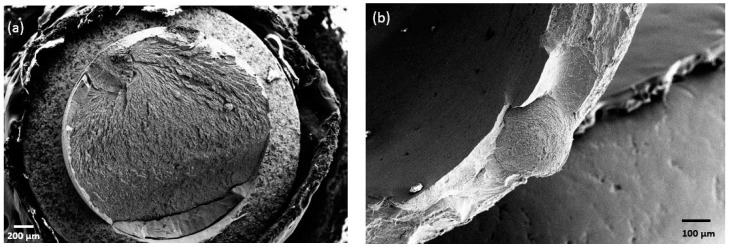
SEM observation of the fracture surface of the Ti_40_Zr_10_Cu_36_Pd_14_ BMG implant-abutment assembly, which broke prematurely during the load to failure test (**a**); and the fatigue fracture surface of an implant made of Ti_40_Zr_10_Cu_36_Pd_14_ BMG (**b**). Large casting defects can be seen.

Figure 12 shows the load as a function of the displacement for three different Ti_40_Zr_10_Cu_36_Pd_14_ BMG implant-abutment prototype assemblies compared to three Ti-6Al-4V alloy implant-abutment assemblies tested under the ISO 14801 standard test. The Ti-based metallic glass exhibits a load to failure 1.5-times higher (450 N) than that of Ti-6Al-4V alloy (300 N) for two of the three tested implants, with a significant plastic deformation before failure. However, the last implant broke at a lower load (but still higher than Ti-6Al-4V) without any plastic deformation. An SEM observation of this sample’s fracture surface is shown in Figure 13. The broken abutment exhibits a large casting defect of approximately 450 µm in diameter, indicating that the premature rupture of the assembly would be a consequence of this metallurgical defect and not due to the nature of the material itself.

**Figure 14:**
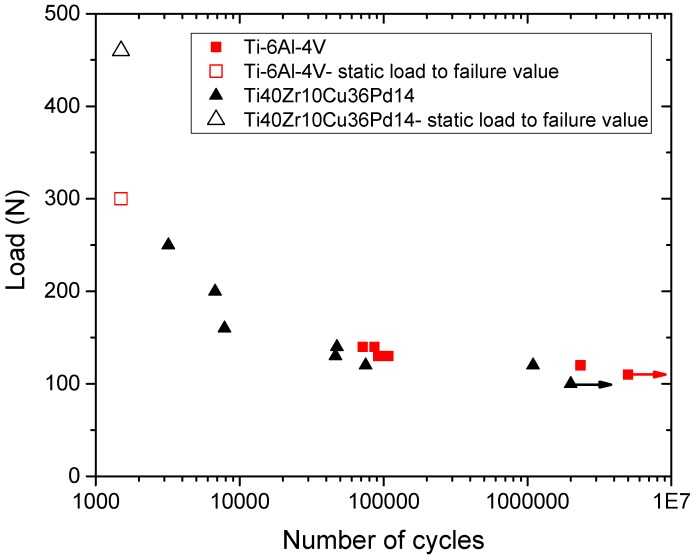
Wölher curve (load as function of the number of cycles) of the Ti_40_Zr_10_Cu_36_Pd_14_ metallic glass compared to the Ti-6Al-4V.

**Figure 15:**
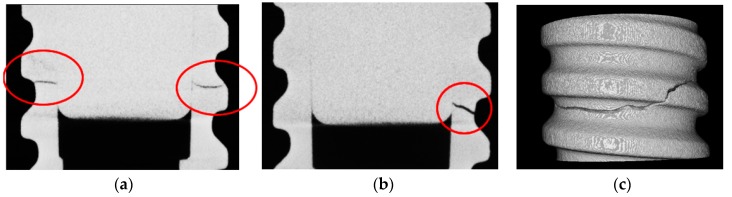
Single slices (**a**,**b**) and 3D view (**c**) of implant and dental abutment assemblies made of Ti_40_Zr_10_Cu_36_Pd_14_ BMG after fatigue test and obtained by X-ray tomography.

Figure 14 shows Wölher curves of the BMG and reference Ti-6Al-4V. The BMG shows a fatigue limit at two million cycles comparable to that of Ti-6Al-4V (100 N). The cyclic fatigue resistance (ratio of fatigue limit to the initial strength) of the BMG (0.22) is therefore lower than that of Ti-6Al-4V (0.33). High resolution X-ray tomography showed that the fatigue crack initiation starts inside the implant, in the last thread, where the stresses are maximal, as for Ti-6Al-4V dental implants (Figure 15). However, the fatigue fracture surface observations performed by SEM (Figure 12) reveal important casting defects (diameter of about 300 µm) inside the structure of the implant. Such casting defects can be responsible for the observed premature failure of one implant during the quasi-static mechanical test and of the relatively low fatigue resistance of the metallic glass.

## 3. Discussion

### 3.1. Glass Forming Ability, Processing Chain and Overall Stability

As seen in Figure 1, the Ti_40_Zr_10_Cu_36_Pd_14_ alloy is fully amorphous to XRD in the form of a 5 mm-diameter rod, which indicates that the alloy exhibits a high glass forming ability allowing one to cast high diameter samples while keeping a fully-amorphous state. This result is in agreement with the literature as previous authors have reported a critical diameter of 6 mm [15]. Machining of dental implants is usually done on bars of approximately 5 mm in diameter. Therefore, such a result is particularly interesting as it may ensure that dental implants could be machined on the Ti_40_Zr_10_Cu_36_Pd_14_ metallic glass rods. For a long time and despite their promises, the use of metallic glasses has been restrained due to the too small achievable dimensions of parts. In that context, dental implantology would be an ideal application as it deals with small-diameter pieces that require very high strength. Besides, the Ti_40_Zr_10_Cu_36_Pd_14_ BMG exhibits a relatively high ΔT_x_ of 38 °C (Figure 2 and Table 1) and a reasonable time before the onset of crystallization at 420 °C (Figure 3). Such results are of prime importance as they evidence the potential of the alloy for shaping processes around T_g_. Easy and economic forging process or surface texturization [34,35,36] could be for example possible at 420 °C while maintaining an almost fully amorphous state.

An interesting point from a practical processing point of view is that the BMG material was machined as easily as the Ti-6Al-4V alloy, as the protocol of machining (turning tools, machines used and machining time) for the two alloys was exactly the same (Figure 10). However, we can note that for the same final surface treatments (i.e., sandblasting and acid washing), the metallic glass shows a slightly lower roughness (1.3 µm) than the Ti-6Al-4V alloy (2.1 µm), as it is harder than its crystalline counterpart alloy. However, this roughness value lies in the recommended range for osteo-integration [37,38] of dental implants and can be easily modified by changing the sandblasting procedure.

As shown in Figure 11, machining of the implant and the abutment pieces made of Ti_40_Zr_10_Cu_36_Pd_14_ alloy has led to a local heating of the alloy, inducing partial surface crystallization. However, after the few first micrometers, the crystallization at the surface of the machined alloy is no longer visible. The phases formed at the surface are intermetallic compounds, which is consistent with the phases formed by simply heating the alloy during the in situ XRD experiment (Table 1). By using the Debye–Scherrer equation (Equation (3)), a crystallite size of 7 nm is found. In this study, the protocol of machining was designed to be the same as the one of Ti-6Al-4V alloy, inducing localized heating of the BMG samples, leading to crystallization at the surface. However, this crystallization was not responsible for the earlier break of some of the samples, which broke because of the presence of large casting defects (Figure 13). Therefore, the optimization of the protocol of machining (decrease of the speed for example) of the metallic glass may lead to the suppression of this thin crystalline layer at the surface. Moreover, further investigation would be necessary to better investigate the features of this partially recrystallized layer, how it is modified by other surface mechanical treatments and how this may play a role (or not) in the properties of BMGs, even if this phenomenon seems to be secondary. In any case, bulk Ti_40_Zr_10_Cu_36_Pd_14_ alloys can withstand the processing chain required for the production of dental implants.

Medical device companies and hospitals use most often two ways of sterilization: steam sterilization at 134 °C or gamma rays. Therefore, these two different types of sterilization were used in this study. The amorphous nature of the designed alloy is not influenced either by the heating at 134 °C or by the gamma ray exposure (Figure 1). The first result is not surprising as the temperature used to sterilize medical materials (134 °C) is much lower than the glass transition and crystallization temperatures of the tested alloy (394 °C and 432 °C, respectively). Concerning the sterilization by gamma irradiation, our result is consistent with previous studies highlighting no effect of gamma rays on the mechanical [39], electrical [40] and magnetic [41] properties of several BMG materials. This proves that sterilization can be performed on Ti_40_Zr_10_Cu_36_Pd_14_ alloy without any deleterious influences regarding their amorphous nature and their future use as implants.

### 3.2. Mechanical Performances

As summarized in Table 2, the Ti_40_Zr_10_Cu_36_Pd_14_ BMG has a Young’s modulus equivalent and even relatively lower (96 GPa) than that of Ti-6Al-4V alloy (104 GPa). Therefore, stress shielding effects would be equivalent or slightly lower than for polycrystalline titanium.

Table 2 shows that the metallic glass exhibits a very high hardness value (556 ± 5 HV_1_), much higher than that of Ti-6Al-4V (341 ± 7 HV_1_). This result is in accordance with the values found in the literature for quite similar alloys [23,32,33]. Such an excellent hardness shows that the Ti-based BMG should lead to a higher elastic limit than the Ti-6Al-4V alloy. The simple assumption, well accepted for a purely elasto-plastic material, that H_v_ = 3σ_y_, would lead to a yield stress of 1850 MPa. This is consistent with the value experimentally obtained (i.e., 1930 MPa) from the compression tests (see Figure 5). Furthermore, no cracks were observed after indentation, even at high load of 30 kgf (Figure 4). This reflects that the metallic glass does not behave as a brittle material, which would be a first proof that the BMG would have a high toughness. As shown in Figure 5, the alloy exhibits a high compressive strength (~2 GPa), with the presence of some ductility (~0.7%), highlighting plasticity before failure. Moreover, the compressive tests are reproducible, and the standard deviation regarding the maximum compressive strength values is really low (see Table 1). Nowadays, several companies are developing new alternatives to Ti-6Al-4V dental implants. Among these solutions, Yttria-Stabilized Tetragonal Zirconia Polycrystal (YTZP) ceramics have gained increasing interest the past few decades [42,43]. However, YTZP ceramics evidence a brittle behavior, which has induced sometimes the sudden breaking of implants. Therefore, the Ti_40_Zr_10_Cu_36_Pd_14_ metallic glass could appear also as a credible alternative to Y-TZP ceramic implants thanks to its more ductile behavior.

The dominant fractographic feature shown in Figure 5 is a smooth and well-developed vein pattern, commonly observed for BMGs and caused by viscous flow of matter in the shear band [21]. X.K. Xi et al. have established a correlation between the length scale of the plastic process zone, known as the vein pattern, and the toughness of BMGs [42]. By evaluating the vein pattern size, w, and using Equation (3), it is possible to estimate the fracture toughness of the material by:

(4) $$w=0.025{\left(\frac{K_{Q}}{\sigma_{y}}\right)}^{2}$$

with *w* the average width of the dimple (or the wavelength of the vein features), *K_Q_* the fracture toughness evaluated from the size of vein patterns and *σ_y_* the yield stress. The results are summarized in Table 3 and compared to the values from the literature [21,42].

Results show that the alloy exhibits high fracture toughness (56 MPa.√m), comparable to the values of the literature, with a relatively large vein pattern. This result is consistent with the fact that no cracks were observed after Vickers indentation at high load (Figure 4). From the knowledge of the toughness and yield stress, it is often proposed to estimate the size of the critical flaw, above which the behavior of a given material tends to become brittle [43]:

(5) $$a_{c}=\frac{K_{Q}{}^{2}}{\sigma_{y}{}^{2}\cdot \pi }$$

with *a_c_* the critical flaw size (above which the material tends to be brittle), *K_Q_* the toughness in MPa.√m and *σ_y_* the yield stress (in MPa). In our case, *K_Q_* was approximated to 56 MPa.√m by means of the vein pattern measurement (Equation (3)), and *σ_y_* is equal to 1930 MPa (Table 2). Therefore, *a_c_* is roughly equal to 270 µm. This is a rough estimation, but it gives an idea about the critical flaw size, above which an implant would fracture before yield.

As far as implants are concerned, the prototypes made with BMG offered great superiority to that made with Ti-6Al-4V alloy for both of them, while one broke at a significantly lower load, without plastic deformation. This later behavior was easily related to a large casting defect of 450 µm, which is higher than the critical defect size estimated above. Such defects, trapped during the tilt-casting, are commonly observed in cast metallic glasses, as reported by S. Yamaura et al. [31]. These defects were not detected by X-ray tomography, because they exhibit the same composition as the surrounding material. The metallic glass exhibits a fatigue limit similar to that of Ti-6Al-4V. However, as the alloy shows an extremely high load to failure (1.5-times higher than that of Ti-6Al-4V), one would have expected a higher fatigue limit. As shown in Figure 13, the lower fatigue properties may be due to large casting defects on the order of 285 µm, higher than the estimated critical flaw size (270 µm). However, it is worth noting that despite the casting defects, the fatigue results are remarkably not dispersed.

The BMG prototype implants thus show good mechanical properties, limited by the presence of randomly-dispersed casting defects, even if the material is highly flaw-tolerant. A better control of the casting defects in metallic glasses is a challenge, and efforts are being conducted today to develop alternative processes such as suction-casting [44,45,46]. This could still allow increasing drastically its mechanical and fatigue properties.

### 3.3. Corrosion Resistance and Cytocompatibility

Excellent corrosion resistance is an essential property for biomedical materials used as long-term implants as they have to withstand the aggressive human body environment without a detrimental release of corrosion-induced metallic-ions, which may yield adverse effects on the host body [37]. Ti_40_Zr_10_Cu_36_Pd_14_ metallic glass exhibits a higher corrosion potential (−0.07 V/SCE) than Ti-6Al-4V alloy (−0.2 V/SCE), as shown in Table 2. This indicates that more energy is required to initiate the corrosion reaction for the BMG. In addition, the corrosion current density of Ti_40_Zr_10_Cu_36_Pd_14_ metallic glass (~ 6 × 10^−9^ A/cm^2^) is in the same order of magnitude as that of Ti-6Al-4V alloy (~ 1.5 × 10^−8^ A/cm^2^), which is known as a reference for its good corrosion behavior. As a consequence of its passive layer, the Ti-based glassy alloy, however, suffers from pitting corrosion at around 500 mV/SCE, as shown in Figure 6. XPS results indicate that the passive layer of the metallic glass is mainly composed of titanium oxide, TiO_2_, and zirconium oxide, ZrO_2_, due to the segregation of Ti and Zr to the surface in the presence of oxygen [47], and its thickness can be estimated at around 15 nm (Figure 7). This passive layer appears highly protective and dense, as reflected by the high polarization resistance of Ti_40_Zr_10_Cu_36_Pd_14_ metallic glass (2.0 MΩ/cm^2^) compared to the reference Ti-6Al-4V alloy (0.5 MΩ/cm^2^). However, a very low content of copper oxide Cu_2_O was detected by XPS at the passive layer surface (Figure 7), which corroborates recent results on binary PVD Zr-Cu coatings [48]. Cu is a detrimental alloying element known to decrease drastically the corrosion resistance of metallic alloys and metallic glasses [49,50,51]. Other studies performed on TiZrCuPd BMGs have revealed the same behavior, with an apparent corrosion resistance with high corrosion potential and low corrosion densities compared to commercial Ti-6Al-4V alloy, but with pitting at potentials around +400–+600 mV/SCE [20,23,32,52]. However, for some authors, such as J.J. Oak [52], an increase in the current density of about 500–600 mV/Open Circuit Potential (OCP) is not a real issue because, in general, human cells and organs use bioelectricity with an appropriate variation range of 70–90 mV. As an example, they mention the fact that the Ti-45 mass % Ni, which is used as a biomaterial in the human body, exhibits an even lower passivation limit of about +250 mV before pitting [53]. Moreover, the current density of the Ti_40_Zr_10_Cu_36_Pd_14_ metallic glass below the breakdown point is lower than that of other Ti-Ni alloys, already extensively used as biomaterials [54,55,56,57].

As shown in Figure 8, independent of the cell types, the viability and proliferation of cells cultured on the Ti_40_Zr_10_Cu_36_Pd_14_ BMG are completely comparable to those on the Ti-6Al-4V alloy. This indicates that the two metallic tested materials have no deleterious effects on cell proliferation.

Moreover, the cells presented similar typical irregular and polygonal shapes with long and thin filopodia (indicated by arrows in Figure 9), which are essential for cell migration and adhesion. As the cell spreading was quite extensive, with numerous pseudopodia visible with distinct anchoring plates, it indicates a satisfactory adhesion. All along the time, cell colonization was observed on the two tested substrates showing a satisfactory cytocompatibility of both Ti_40_Zr_10_Cu_36_Pd_14_ BMG and Ti-6Al-4V alloy. We can note that the morphology of cells grown on the Ti-based BMG is similar to the case of Ti-6Al-4V substrates, as well as to the findings in previous works for other BMGs [32,33,58,59,60,61].

In terms of biocompatibility, the presence of Cu and Pd might be questionable. However, these elements are part of the structure of the alloy and not present as free atoms. Moreover, in contrast with Ni and Be, which are known to be toxic, Cu, quite often, is part of medical devices due to its antibacterial properties, and both elements have been previously included in alloys without any adverse effects, as shown in previous studies [62,63,64].

### 3.4. Limits of the Study and Further Work

Ti_40_Zr_10_Cu_36_Pd_14_ metallic glass exhibits very interesting mechanical properties compared to the well-known Ti-6Al-4V reference alloy (Table 2). However, in this study, the static mechanical properties on dental implant-abutment assemblies’ prototypes were only analyzed on a limited number of samples (three different samples for the static tests and eight samples for the fatigue ones), as the processing of the metallic glasses was done on a limited amount of material. Therefore, in order to draw a more statistical trend of the mechanical properties of the studied Ti-based alloy, further tests would be necessary with a more realistic and large number of samples in order to add a statistical analysis for all the properties measured.

Moreover, large casting defects were observed inside the implant-abutment prototypes, which have led to lower mechanical properties. In this context, there is a need to better control the processing of the Ti_40_Zr_10_Cu_36_Pd_14_ for the industrial development of dental pieces on a wider scale in order to avoid any premature failure of the assemblies.

The machining of Ti_40_Zr_10_Cu_36_Pd_14_ dental implants following the same machining conditions as Ti-6Al-4V has led to the crystallization of a thin layer at the surface (Figure 11). However, the nature and impact of such a crystalline layer at the surface was not investigated in detail in this study. Further investigation would be necessary to understand how this crystalline layer may influence the mechanical properties of the alloy. Refining the machining strategy could be an option to suppress this crystalline layer if necessary. The studied Ti-based metallic glass exhibits a high resistance to corrosion with a corrosion potential superior to that of Ti-6Al-4V alloy. Nevertheless, in this study, the electrochemical measurements were performed only in a 0.9% NaCl electrolyte, following the ISO-10271 standard. Regarding the future potential implantation of the alloy, further corrosion tests would be necessary in more aggressive media, to better mimic conditions found in the human body environment. Previous authors have found that media containing H_2_O_2_ and albumin could accelerate the corrosion damages, even of highly corrosion-resistant alloys such as titanium ones [65,66]. Such corrosive media could be a good alternative to 0.9% NaCl electrolyte to perform new electrochemical tests. In this regard, the long-term stability of the developed Ti-based BMG should be validated, as no pitting at low potential values could be tolerated for an implantable device, especially in an aqueous environment similar to the human body.

After all, static immersion tests and Inductively Coupled Plasma (ICP) measurements would be necessary to complete the in vitro results performed in this study, in order to validate that no potentially toxic ion-release is observed. Moreover, further in vivo assessments should be performed to ensure that no deleterious effects follow the implantation of Ti_40_Zr_10_Cu_36_Pd_14_ material.

## 4. Materials and Methods

### 4.1. Materials Characterization

#### 4.1.1. Materials

Ingots with a targeted composition of Ti_40_Zr_10_Cu_36_Pd_14_ (at %) were prepared by arc melting the pure elements (purities above 99.9%). Rods with a diameter of 5 mm were prepared using a tilt copper mold-casting apparatus in a high purity argon atmosphere. The composition of all the ingots was controlled by Energy-Dispersive X-ray spectroscopy (EDX) on a Scanning Electron Microscope (SEM) (Zeiss Supra 55, Oberkochen, Germany) at an acceleration voltage of 15 keV. Their density was measured by the Archimedes method.

#### 4.1.2. Structural Analysis

The amorphous nature of all the BMGs’ samples after processing was first estimated by X-ray Diffraction (XRD) (Bruker AXS D8 advance, Karlsruhe, Germany) analysis, using Cu-Kα radiation. The kinetics of crystallization were then studied by High Temperature X-ray Diffraction (HTXRD), either during in situ heating or versus time at a given temperature of 420 °C. Experiments were performed using an Anton Paar HTK 1200 oven chamber, under vacuum. The heating rate was fixed to 0.75 K/min, and diffractograms were recorded in the 2θ region ranging from 35°–50°. Such a heating rate was chosen in order to find the proper compromise between accuracy and a sufficient scan speed to compare with the Differential Scanning Calorimetry (DSC) scans (performed at a heating rate of 20 K/min). Diffractograms were recorded either during continuous heating every 5° or every 7 min during isothermal annealing at 420 °C.

Grazing incident X-rays were used with a Goeble mirror to investigate a potential crystallization at the surface of the machined pieces. The 2θ angles range was 20°–80° with an incidence angle of 5°, an increment of 0.05 and a step size of 15 s. The calculated penetration depth, using the measured density of the alloy and the AbsorbX software, was approximately 1.4 µm. The XRD pattern obtained in grazing incidence was compared with the same spectra acquired in the Bragg–Brentano configuration (penetration depth at 2θ = 80° of 9 µm). Comparison of both diffractograms, acquired in grazing incidence or in the Bragg–Brentano configuration, will indicate localization of the crystallization initiation, either throughout the material or only at its top surface.

Prior to the mechanical characterization of the BMG samples and to confirm the good repeatability of the samples’ process, the quality of the material (absence of large porosities or gradients of composition) was characterized by X-ray tomography on 3 mm-diameter rods. Fast acquisitions, at low resolution, were carried out using a Vtomex device (GE Phoenix|X-ray GmbH, Boston, MA, USA) equipped with a 160-kV nano-focus tube, a tungsten transmitting target and a 1920 × 1536 pixel Varian detector; see [67] for more details. A current of 60 μA was used with a 0.1-mm copper filter, using a voxel size of 30 µm^3^.

X-ray tomography was also used to perform 3D analysis at high resolution (4 µm^3^ per voxel) to characterize the mechanisms of failure and crack initiation of prototype implants. For this high-resolution analysis, another device (RX solution) was used. The Hamamatsu X-ray source was operated at a voltage of 160 kV and a current of 100 μA with a 0.1-mm copper filter. The detector was a Hamamatsu 2300 × 2300 pixels flat panel.

All the cone-beam X-ray computed Tomography (XCT) data were reconstructed by a filtered back projection Feldkamp algorithm, processed and visualized with the Image J software [68,69].

#### 4.1.3. Thermal Stability

Glass transition temperature (T_g_) and crystallization temperature (T_x_) were characterized by Differential Scanning Calorimetry (DSC) at a heating rate of 20 K/min using a standard commercial instrument (Perkin Elmer, DSC-7, Waltham, MA, USA) under high purity dry nitrogen at a flow rate of 20 mL/min.

#### 4.1.4. Mechanical Characterization

Young’s modulus was determined by ultrasonic measurement, performed on a Wave Runner HRO64Zi Oscilloscope (400 MHz, 12 bits, 2 GS/s) on both the BMG and Ti-6Al-4V alloy disks (3-mm diameter and 5-mm length).

Vickers hardness tests were performed on mirror polished samples (until 1 µm) using two different equipment: a Buehler Micromet 5140 for loads lower than 1 kgf (9.8 N) and a Future-Tech Vickers hardness tester FV-700 for loads up to 30 kgf (29.4 N). The measurements were repeated 5 times on 13 different samples.

Compressive tests were performed on cylinders of 3 mm diameter and 5 mm in length on an INSTRON 5967 testing machine with a 30-kN cell and an optical extensometer. Tests were performed at ambient temperature at a strain rate of 1.7 × 10^−4^ s^−1^. Measurements were done on 6 samples. Fracture surface observations were carried out by SEM, using an acceleration voltage of 15 keV, with the aim of describing fracture mechanisms and observing vein patterns.

### 4.2. Characterization for a Potential Biomedical Application

#### 4.2.1. Resistance to Sterilization

To check that the usual sterilization methods do not influence the glassy character of the material, two types of sterilization techniques were considered:

- Heat treatment in autoclave performed in water steam at 134 °C, under a 2-bar pressure for 20 h (mimicking 20 sterilization cycles),
- Gamma rays (a total dose of 100 kGy was applied by subsequent conventional 25–40 kGy medical devices irradiations).

The amorphous structure of the BMG after both sterilization modes was then confirmed by XRD.

#### 4.2.2. Corrosion Resistance

The corrosion resistance and passivation behavior of Ti_40_Zr_10_Cu_36_Pd_14_ BMG were investigated by electrochemical measurements and compared to the Ti-6Al-4V reference alloy. Electrochemical measurements were conducted in a typical three-electrode glass cell, using a Parstat 2273 potentiostat with graphite as the auxiliary electrode and a Saturated Calomel Electrode (SCE) as reference electrode. Prior to electrochemical measurements, both BMG and reference samples were ground using abrasive papers followed by diamond paste polishing down to 1 µm. Saline solution (0.9% NaCl, pH: 7.4 (ISO 10271)) at 37 °C open to air was used as the electrolyte. This solution was chosen as it is recommended in the ISO 10271 standard, dedicated to the corrosion test methods for metallic materials used in dentistry. As the passivity of an alloy is directly affected by the presence of chloride ions, saline solution is an adequate electrolyte to measure such a property. Before potentio-dynamic polarization measurements, OCP measurements were performed during 3 h to stabilize the samples in the saline solution. During this step, the polarization resistance was also measured every 30 min at a scan rate of 0.166 mV/s (±10 mV vs. OCP). Finally, linear polarization was performed, with a scan rate of 0.166 mV/s, from −0.25 V (vs. OCP) to +1.6 V (vs. SCE). The corrosion potential (*E_corr._*) and the corrosion current density (*I_corr._*) were estimated by Tafel’s extrapolation method from anodic polarization curves.

The passive oxide film formed on the surface of the BMG was characterized by X-ray Photoelectron Spectroscopy (XPS) using a PHI Quantera SXM photoelectron spectroscopy instrument (Ulvac Inc., Chigasaki, Japan) with an analyzed area diameter of 200 µm. The XPS profiles were obtained after successively realizing acquisition steps and ionic abrasion with Ar ions, at a speed of around 14.3 nm/min. SiO_2_ was used as a reference to estimate the abrasion depth.

#### 4.2.3. Cytocompatibility

Direct cytotoxicity tests were carried out using MG63, osteoblast-like cells (Cell Culture Passage Number 45) (ATCC, Ref CRL-1427) and adult Human Dermal Fibroblasts (HDFa) (Cell Culture Passage Number 9) (obtained from adult skin explants) to evaluate cell proliferation and adhesion after 3, 6 and 10 days of culture. Cells were cultured in 24-well cell culture plates (Corning, NY, USA, ref 3524) with RPMI 1640 medium (Dutscher, France, ref L0498-500) (containing stable L-glutamine and phenol red) supplemented with 10% fetal bovine serum (Dutscher, ref P040637100) and 5% antibiotic/antimycotic solution (Dutscher, Ref SV30079.01) at 37 °C in a humidified atmosphere of 5% CO_2_. Two different alloys were tested; the Ti-6Al-4V alloy (biomedical grade) used as a reference and the Ti_40_Zr_10_Cu_36_Pd_14_ BMG as the studied material were compared to a control group (culture dish surface; polystyrene treated for cell culture). The samples were discs of 1 mm thick and 5 mm in diameter. The metallic discs were sterilized by immersion in ethanol (70%) for 20 min and dried.

A cellular suspension at 100,000 cells/mL was prepared using a cell counter (Millipore Scepter purchased at Dutscher, ref: 053750). The Scepter cell counter uses the Coulter principle of impedance-based particle detection to reliably and accurately count every cell in the sample.

After sterilization, the discs were placed in the 24-well cell culture plates (one sample in each well). Then, 50 μL of cellular suspension were deposited at the surface of the materials (5 × 103 cells per sample), and cell culture plates were incubated 2 h at 37 °C under a humidified atmosphere of 5% CO_2_ to allow cell adhesion. After the addition of 2 mL of culture medium, cells were incubated until 10 days.

The cell viability was measured using the PrestoBlue technique (Invitrogen, Carlsbad, CA, USA) [70]. The resazurin, which is blue and non-fluorescent, is reduced by the metabolic mitochondrial activity of the cells in resorufin, a pink and fluorescent product, easily detectable by fluorometry. After 3, 6 and 10 days, the culture medium was discarded and replaced by 1 mL of the culture medium without FBS, antibiotics and phenol red, but with 10% PrestoBlue put on each well. The samples were incubated 1.5 h at 37 °C under a humidified atmosphere of 5% CO_2_. Then, the plate was stirred, and 100 μL of each well were transferred in a 96 black-well plate and the fluorescence measured using an INFINITE PRO 200 fluorimeter (Tecan) with a wavelength of 535 nm for excitation and 615 nm for emission. After each measurement, cells were rinsed two times with RPMI, then 2 mL of medium were added and the plate incubated until the next measure (6 and 10 days). Each assay was done in triplicate.

To evaluate the cell morphology and attachment, the samples were observed by SEM (Hitachi S800 FEG, 15 kV, Chiyoda, Japan) after 6, 24 and 72 h of incubation. The same procedure as the one used to evaluate the cell viability was used (cells culture, sterilization of the samples, deposition of the samples in the wells, addition of the cellular suspension, incubation and addition of the culture medium). After the incubation, the cells were fixed on the surface disks with 2% glutaraldehyde, dehydrated progressively, metallized by Au/Pd, and then observed by SEM.

### 4.3. Characterization of Implant-Abutment Assemblies

#### Tests on Dental Implants

Mechanical tests on real-sized dental implant and abutment assemblies were performed on BMG samples after machining, following the same machining conditions as real medical-grade Ti-6Al-4V implants, including a turning step on a Computer Numerical Control (CNC) lathe and sandblasting with a resorbable blasting media (Hydroxyapatite/TriCalciumPhosphate (HAP/TCP) mixture), followed by acid washing in HNO_3_. The same machining and sandblasting conditions were used for both BMG and Ti-6Al-4V. The roughness parameter (Sa) of both alloys was then measured by white light vertical scanning interferometry (vsi), with a Sensofar Neox machine, using an objective of 50×.

Mechanical characterization and evaluation of the load to failure on narrow diameter dental implants (Ø 2.8 mm) and abutment assemblies were evaluated on a BOSE ELF3300 testing machine (New Castle, PA, USA) following the geometrical specifications of ISO 14801 (displacement rate of 5 mm/min). Fatigue was performed using the same machine and geometry, in 0.9% NaCl solution at a frequency of 2 Hz with a sinusoidal load between 0.1- and 1-times the maximum applied load, up to failure or 2 × 10^6^ cycles. Results were compared to Ti-6Al-4V implant-abutment assemblies with the same geometry and machining conditions.

According to ISO14801-2007 prescriptions, implants were embedded in a resin having a Young’s modulus of 7 GPa after polymerization (Rencast© Resin, HUNTSMAN) and with 3 mm of non-impregnated threads simulating bone resorption. Abutments were then fixed on the implants according to the manufacturer’s instructions. Hemispherical caps were seated onto abutments in order to ensure a constant lever arm of 11 mm on each assembly for mechanical testing. The fatigue fracture surfaces were again observed by SEM in order to identify the fracture initiation and behavior.

## 5. Conclusions

In summary, Ti_40_Zr_10_Cu_36_Pd_14_ bulk metallic glass evidences a high glass forming ability allowing it to be cast in a 5 mm-diameter rod shape, while keeping its fully amorphous structure. The sample diameter is suitable for the classical processing chain of dental implants, as it deals with small dimension pieces. Moreover, the Ti-based BMG is machinable and can be sandblasted without damage, with protocols currently used for titanium. It has shown to be highly stable in temperature, which allows it to be thermally processed easily and suitable for biomedical sterilization. Ti_40_Zr_10_Cu_36_Pd_14_ BMG passivates spontaneously and forms a dense passive film, which leads to a good corrosion resistance with a higher corrosion potential and a lower corrosion rate than Ti-6Al-4V alloy. Moreover, it shows a satisfactory cytocompatibility, with a cell morphology, viability and adherence comparable to that of Ti-6Al-4V alloy. Finally, this metallic glass exhibits excellent mechanical properties, exceeding those of the Ti-6Al-4V alloy.

In this regard, Ti_40_Zr_10_Cu_36_Pd_14_ metallic glass appears as a viable alternative to Ti-6Al-4V alloy for the manufacturing of implant and abutment assemblies or small dimension pieces for future dental applications.

## Figures and Tables

**Table 1:** Chemical composition and thermal properties of the Ti_40_Zr_10_Cu_36_Pd_14_ Bulk Metallic Glass (BMG).

Chemical Composition (At %)				Thermal Stability			Phases and Crystallization Temperature (°C)		
Ti	Cu	Pd	Zr	T_g_ (°C)	T_x_ (°C)	ΔT_x_ (°C)	PdTi_2_	Cu_3_Zr_8_	CuTi
40.2	36.4	14	9.4	394	432	38	450	450	450

**Table 2:** Electrochemical and mechanical properties of the Ti_40_Zr_10_Cu_36_Pd_14_ BMG compared to the Ti-6Al-4V alloy.

Alloy	Corrosion Properties			Mechanical Properties						
	*E_corr._* (V/SCE)	*I_corr._* (A/cm^2^)	*R_p_* (MΩ/cm^2^)	*E* (GPa)	Hardness (HV_1_)	*σ_m_* ^1^ (GPa)	*σ_y_* ^2^ (MPa)	*ε_el_* (%)	*ε_p_* (%)	*σ_s_* ^3^ (N∙m/kg)
BMG	−0.07	~6 × 10^−9^	2.0	96	556.4	2.01	1930	2	0.73	2.8 × 10^5^
Ti-6Al-4V	−0.2	1.5 × 10^−8^	0.5	115	341	0.97	1050	1	10	2.0 × 10^5^

^1^ Compressive strength. ^2^ Yield strength. ^3^ Specific strength.

**Table 3:** Data of measured *w*, *K_Q_* and *σ_y_* of the Ti_40_Zr_10_Cu_36_Pd_14_ BMG compared to the values from the literature.

Metallic Glasses	*w* (µm)	*K_Q_* (MPa.√m)	*σ_y_* (MPa)	Reference
Ti_40_Zr_10_Cu_36_Pd_14_	19.5 (±6.3)	56	2010 (±15)	Original
Ti_40_Zr_10_Cu_36_Pd_14_	20.3	55.6	1950	[15]
